# A nationwide cohort study for comparative vascular safety of long-acting insulin analogue versus intermediate-acting human insulin in type 2 diabetes

**DOI:** 10.1038/s41598-021-83253-6

**Published:** 2021-02-18

**Authors:** Chun-Ting Yang, Kuan-Ying Li, Chen-Yi Yang, Huang-Tz Ou, Shihchen Kuo

**Affiliations:** 1 Institute of Clinical Pharmacy and Pharmaceutical Sciences, College of Medicine, National Cheng Kung University, 1 University Road, Tainan, 701 Taiwan; 2 Department of Pharmacy, College of Medicine, National Cheng Kung University, Tainan, Taiwan; 3 Department of Pharmacy, National Cheng Kung University Hospital, Tainan, Taiwan; 4 Division of Metabolism, Endocrinology & Diabetes, Department of Internal Medicine, University of Michigan Medical School, Ann Arbor, MI USA

**Keywords:** Diseases, Endocrinology, Medical research

## Abstract

Little is known about the comparative vascular safety of basal insulins (intermediate-acting human insulin [IAHI] or long-acting insulin analogue [LAIA]) in type 2 diabetes (T2D). This study sought to examine the vascular and hypoglycemic effects associated with IAHI versus LAIA in real-world patients with T2D. We utilized Taiwan’s National Health Insurance Research Database to identify T2D patients who stably used IAHI (N = 11,521) or LAIA (N = 37,651) in the period 2004–2012. A rigorous three-step matching algorithm that considered the initiation date of basal insulin, previous exposure of antidiabetic treatments, comorbidities, diabetes severity and complications, and concomitant medications was applied to achieve the between-group comparability. Study outcomes, including cardiovascular diseases (CVDs), microvascular diseases (MVDs), and hypoglycemia, were assessed up to the end of 2013. Compared with LAIA, the use of IAHI was associated with greater risks of composite CVDs (adjusted hazard ratio [aHR]: 1.79; 95% confidence interval [CI] 1.20–2.67) and hospitalized hypoglycemia (aHR: 1.82; 95% CI 1.51–2.20), but a lower risk of composite MVDs (aHR: 0.88; 95% CI 0.84–0.91). Subgroup and sensitivity analyses showed a consistent trend of results with that in the primary analyses. In summary, although the use of IAHI versus LAIA among T2D patients in usual practice may be associated with a lower risk of MVDs, strategies should be optimized for minimizing the risks of hypoglycemia and CVDs in this population.

## Introduction

According to the American Diabetes Association’s Standards of Medical Care in Diabetes—2020, basal insulin including intermediate-acting human insulin (IAHI) and long-acting insulin analogue (LAIA), is suggested for patients with type 2 diabetes (T2D) who require initiation of insulin therapy^1^. Owing to the reduced risk of symptomatic and nocturnal hypoglycemia of LAIA versus IAHI as demonstrated in clinical studies^2–5^, LAIA is generally preferable in clinical practice compared to IAHI, such as human neutral protamine Hagedorn (NPH) insulin. However, because of the comparable efficacy of glycemic control between LAIA and IAHI^2,6^ and the lower drug acquisition cost of IAHI, initiation with IAHI or a switch from LAIA to IAHI may be considered for the patients with a low risk of hypoglycemia, prominent insulin resistance, or cost concerns^1,7^.

Evidence on the long-term comparative vascular safety associated with the use of basal insulin in a real-world population with T2D remains limited and shows inconclusive results^8–10^. Moreover, these studies were based on the incident new-user cohort design and thus only included the naïve users of basal insulins. This would limit the generalizability of study findings to real-world settings where some patients initiated with LAIA have been previously exposed to IAHI (i.e., prevalent new users of LAIA), or vice versa. In addition, due to clinical inertia in the management of T2D, basal insulin is commonly not initiated until the later course of antidiabetic treatment, that is, when treatment with multiple oral glucose-lowering agents (GLAs) has failed^11^. Thus, a rigorous analytic scheme is required to address the complexity of past utilization of GLAs for studies assessing health outcomes associated with the LAIA and IAHI use. Furthermore, due to the progressive nature of T2D, many patients eventually require and benefit from insulin therapy, and thus sound evidence on the long-term effects of basal insulin is needed for optimizing clinical diabetes care.

Against this background, we sought to investigate the long-term vascular safety of basal insulin (IAHI versus LAIA) using a large nationwide longitudinal diabetic cohort and a rigorous prevalent new-user cohort design to include a broad representation of real-world adults with T2D being treated with basal insulin. The prevalent new-user cohort design is used to ensure the comprehensiveness and generalizability of study results to clinical practice settings^12^.

## Methods

### Data source

This is a retrospective cohort study utilizing Taiwan’s National Health Insurance Research Database (NHIRD) 1999–2013. The NHIRD is a population-based database derived from the claims data of the National Health Insurance (NHI) program, which is a mandatory-enrollment, universal healthcare system that covers over 99% of Taiwan’s approximately 23 million citizens. The NHIRD provides de-identified longitudinal medical and prescription information for each enrolled beneficiary^13^. The study was approved by the Institutional Review Board of National Cheng Kung University Hospital (B-EX-103-015). The need for the informed consent was waived by the Institutional Review Board of National Cheng Kung University Hospital. The study was carried out in accordance with relevant guidelines and regulations.

### Cohort identification

**Figure 1:**
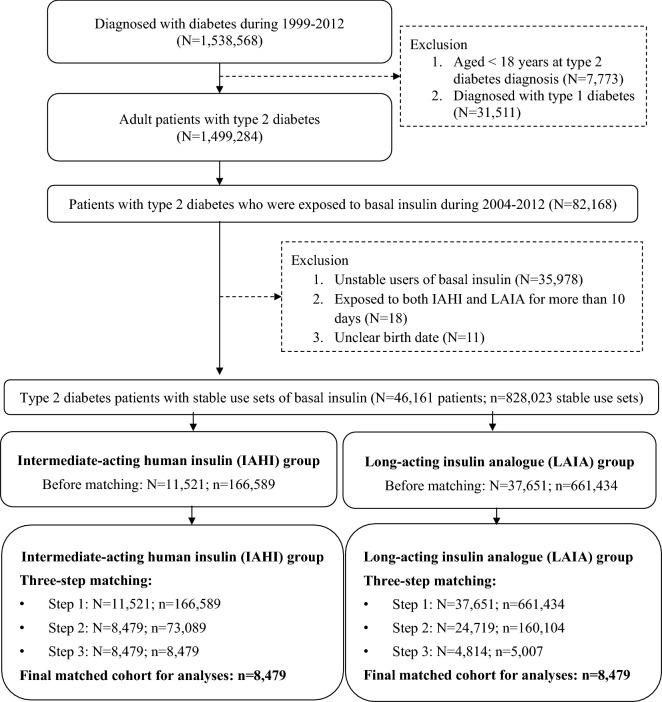
Flowchart of the study population selection and identification. (1) “N” represents the number of patients, and “n” represents the number of stable use sets. (2) Matching steps: (1) Step 1: matched by the cohort entry time (i.e., matching of the index date between the two study drug groups; the index date refers to the first date of IAHI or LAIA use). (2) Step 2: matched by the utilization pattern of glucose-lowering agents within 1 year prior to the index date. (3) Step 3: matched by the propensity score estimated based on patient characteristics measured within 1 year prior to the index date.

Patients with newly diagnosed T2D were identified during the period of 1/1/1999 to 12/31/2012 if they met one of the following criteria: (1) at least one inpatient diagnosis of T2D (International Classification of Diseases, Ninth Revision, Clinical Modification [ICD-9-CM]: 250.X0 or 250.X2, where X = 0–9), (2) at least two outpatient diagnoses of T2D within the same year, or (3) at least one outpatient diagnosis of T2D and with prescription of GLAs in the same year. Patients who were diagnosed with type 1 diabetes or aged < 18 years at the diagnosis of T2D were excluded. Next, to avoid potential confounding from short-term or accidental use of IAHI (study drug) or LAIA (comparator drug), we only included stable drug users in the analyses. Specifically, stable users are those having at least one stable use set of IAHI or LAIA, which was defined as at least three consecutive refills of IAHI or LAIA with any gaps between two consecutive refills of less than 30 days, during the period of 1/1/2004 to 12/31/2012. A stable user of IAHI or LAIA can thus have multiple stable use sets of that drug chronologically. For each stable use set, the first date of IAHI or LAIA use was defined as the index date, and it was followed until treatment discontinuation (i.e., having no consecutive drug refills of the study drug or a refill gap of more than 30 days between two refills), the occurrence of study outcomes, lost to follow-up in the NHI program, death, or the end of the database (i.e., 12/31/2013), whichever came first. The period 2004–2012 was chosen for identifying stable users of the study drugs because LAIA was not reimbursed by Taiwan’s NHI program until 2004, and 2012 allowed a follow-up period of at least 1 year. The flowchart of study cohort selection is shown in Fig. 1.

### Matching algorithm

We applied a three-step matching algorithm to enhance the comparability of baseline patient characteristics between two study group (Supplementary Figure 1)^12,14^. To keep as many IAHI users (the study drug group) as possible in the analyses and match them to the most comparable LAIA users (the comparator drug group) with similar baseline patient characteristics, the stable use sets from the LAIA group were re-used through the matching process.

We first aligned the cohort entry time of the study groups to avoid differences in time-related biases and confounding effects that arise from the evolution of clinical treatment and practice over time. For each stable use set of IAHI, we identified the LAIA stable use set with an index date falling within ± 180 days of the index date of the IAHI stable use set. Then, because the utilization patterns of GLAs prior to the IAHI or LAIA use could be an important indicator of diabetes progression and severity, we adjusted for the history of past GLA use, including metformin, sulfonylureas, meglitinides, thiazolidinediones, acarbose, dipeptidyl peptidase-4 inhibitors, glucagon-like peptide-1 receptor agonists, short-acting/rapid-acting insulins, IAHI, LAIA, and premixed insulins, within the year before the index date. The previous 1-year GLA utilization patterns for the matched pairs of IAHI and LAIA users had to meet two criteria: (1) being exposed to the same GLA classes, and (2) having a difference in the total number of days’ supply of less than 90 (± 45 days) for each specific GLA. Lastly, one-to-one 8-digit greedy propensity score (PS) matching was used to adjust for other baseline patient characteristics (e.g., demographics, diabetes-related complications, comorbidities, and other medication use; Table 1) between the two study groups.

**Table 1:** Baseline patient characteristics before and after application of the matching algorithm.

Characteristics	Before matching			After matching		
	IAHI	LAIA	SMD	IAHI	LAIA	SMD
Number of stable use sets	166,589	661,434		8479	8479	
**Year of index date (%)**			0.87			0.04
2004	4.49	0.04		1.72	1.44	
2005	5.65	0.32		3.88	3.90	
2006	10.31	1.08		10.54	9.64	
2007	12.46	3.83		15.37	15.91	
2008	13.05	8.43		15.90	16.22	
2009	13.41	13.74		13.87	14.27	
2010	13.43	19.06		12.87	12.97	
2011	14.20	24.72		13.06	13.00	
2012	13.01	28.77		12.80	12.65	
**GLA utilization pattern at one year prior to index date**						
Number of GLAs prescribed (mean ± SD)	3.09 ± 1.35	3.71 ± 1.23	0.48	2.26 ± 1.27	2.26 ± 1.27	0.00
MPR (mean ± SD)						
Metformin	0.38 ± 0.43	0.63 ± 0.42	0.33	0.39 ± 0.43	0.39 ± 0.44	0.00
Sulfonylurea	0.34 ± 0.42	0.61 ± 0.43	0.34	0.40 ± 0.44	0.40 ± 0.44	0.00
Meglitinide	0.04 ± 0.16	0.10 ± 0.27	0.31	0.02 ± 0.13	0.02 ± 0.13	0.00
Thiazolidinedione	0.07 ± 0.22	0.15 ± 0.30	0.15	0.07 ± 0.22	0.07 ± 0.22	0.00
Acarbose	0.09 ± 0.26	0.17 ± 0.33	0.12	0.06 ± 0.22	0.06 ± 0.22	0.00
Dipeptidyl peptidase-4 inhibitor	0.02 ± 0.13	0.14 ± 0.30	0.19	0.02 ± 0.12	0.02 ± 0.12	0.00
Glucagon-like peptide-1 receptor agonist	0.00 ± 0.00	0.00 ± 0.00	0.00	0.00 ± 0.00	0.00 ± 0.00	0.00
Short-acting/rapid-acting insulin	0.28 ± 0.38	0.06 ± 0.20	1.33	0.13 ± 0.30	0.13 ± 0.30	0.00
Intermediate-acting human insulin	0.64 ± 0.35	0.01 ± 0.08	1.33	0.30 ± 0.40	0.30 ± 0.40	0.00
Long-acting insulin analogue	0.01 ± 0.08	0.66 ± 0.35	1.38	0.03 ± 0.13	0.03 ± 0.13	0.00
Premixed insulin	0.05 ± 0.17	0.03 ± 0.15	0.22	0.05 ± 0.18	0.05 ± 0.18	0.00
**Characteristics for propensity score matching**						
Age at index date (years, mean ± SD)	59.83 ± 13.83	58.85 ± 12.50	0.07	59.04 ± 15.27	57.60 ± 14.43	0.10
Male (%)	53.15	54.88	0.03	52.61	58.97	0.13
Diabetes duration (years, mean ± SD)	6.88 ± 3.16	7.77 ± 2.03	0.33	6.03 ± 3.43	5.82 ± 3.47	0.06
Number of A1_C_ tests (times, mean ± SD)	2.95 ± 2.10	3.73 ± 2.03	0.38	2.39 ± 2.03	2.53 ± 1.99	0.07
Hospital grade (%)			0.01			0.15
Medical center	33.21	32.69		32.88	39.99	
Non-medical center	66.79	67.31		67.12	60.01	
Diabetes-related complications (%)						
Retinopathy	21.20	22.34	0.03	16.20	20.70	0.12
Nephropathy	24.68	24.68	0.00	20.95	22.49	0.04
Neuropathy	19.15	19.00	0.00	16.17	16.74	0.02
Peripheral vascular diseases	6.06	5.29	0.03	5.09	4.76	0.02
Cerebrovascular diseases	20.45	19.62	0.11	13.15	9.40	0.12
Cardiovascular diseases	12.48	9.15	0.02	19.70	18.83	0.02
Metabolic complications	3.47	2.20	0.08	3.96	4.62	0.03
Hypoglycemia	2.63	1.77	0.06	1.88	1.80	0.01
Comorbidities (%)						
Hypertension	57.83	61.28	0.07	52.31	51.30	0.02
Hyperlipidemia	43.41	57.56	0.29	38.68	39.56	0.02
Stroke or transient ischemic attack	5.49	4.15	0.11	13.37	9.56	0.12
Heart failure	1.33	1.33	0.06	6.10	4.71	0.06
Myocardial infarction	15.63	15.64	0.00	1.52	1.33	0.02
Ischemic heart diseases	12.73	9.24	0.00	14.31	14.85	0.02
Diabetic ketoacidosis	1.80	1.19	0.05	1.98	3.02	0.07
Hyperosmolar hyperglycemic state	1.68	1.04	0.06	2.05	1.67	0.03
CIC category (%)						
Cancers	6.88	6.88	0.00	7.89	6.22	0.07
Gastrointestinal diseases	29.90	27.02	0.06	29.44	26.61	0.06
Musculoskeletal diseases	33.87	35.27	0.03	33.03	33.19	0.00
Pulmonary diseases	12.64	9.18	0.11	13.10	10.19	0.09
Substance abuse complexity	2.46	2.22	0.02	3.28	2.59	0.04
Mental illnesses	11.29	10.11	0.04	10.57	9.51	0.04
CVD-related medication history (%)						
Lipid modifying agents	46.12	59.86	0.28	38.86	40.55	0.03
α-blockers	5.83	5.71	0.01	5.26	4.65	0.03
β-blockers	31.97	32.06	0.00	29.07	27.46	0.04
RAAS agents	51.69	59.73	0.16	44.78	44.92	0.00
Diuretics	32.60	25.56	0.16	29.52	28.60	0.02
Calcium channel blockers	42.93	40.77	0.04	38.72	35.92	0.06
Antiarrhythmics	2.45	1.87	0.04	2.55	1.64	0.06
Cardiac glycosides	4.52	2.66	0.10	4.34	3.66	0.03
Vasodilators	14.54	12.22	0.07	12.81	11.90	0.03
Anti-platelets	37.93	39.87	0.04	33.81	33.35	0.01
Anti-coagulants	1.68	1.34	0.03	1.64	1.46	0.01

IAHA, intermediate-acting human insulin; LAIA, long-acting insulin analogue; SMD, standardized mean difference; GLA, glucose-lowering agent; SD, standard deviation; MPR, medication possession ratio; CIC, Chronic Illness with Complexity; CVD, cardiovascular disease; RAAS, renin–angiotensin–aldosterone system.

### Operational definitions of drug exposure and study outcomes

The use of drugs was measured according to the World Health Organization Anatomical Therapeutic Chemical Classification system and the Taiwan Food and Drug Administration drug license codes^15^. The primary study outcome was the composite of cardiovascular diseases (CVDs), which included fatal or non-fatal myocardial infarction, ischemic heart disease, heart failure, cerebrovascular diseases, cardiogenic shock, sudden cardiac arrest, arteriosclerotic cardiovascular disease, and arrhythmia. Secondary study outcomes were (1) microvascular diseases (MVDs) including nephropathy, retinopathy, and neuropathy, (2) hospitalized hypoglycemia, (3) all-cause death, (4) fatal CVDs, and (5) three-point major adverse cardiovascular events (MACE), including non-fatal myocardial infarction, non-fatal stroke, and fatal CVDs. CVDs and hypoglycemia were identified from the inpatient and emergency department records of the NHIRD to ensure that the underlying CVD or hypoglycemia history was not measured as study outcomes, while MVDs were measured from both inpatient and outpatient department records to capture all possible microvascular events which might be treated in ambulatory settings. The mortality status was ascertained from the inpatient department records. The detailed operational information of study outcomes is provided in the Supplementary Table 1. The validity of ICD-9-CM coding for study outcomes measured from the NHIRD is documented elsewhere^16–21^.

### Statistical analyses

Baseline patient characteristics were measured from the year before or at the index date. Differences in baseline patient characteristics between the study groups before and after applying the matching algorithm were compared using the standardized mean difference (SMD), where SMD values of > 0.1 indicate a statistically significant between-group difference^22,23^. The event rates of study outcomes were calculated as the total number of events over the follow-up period divided by the number of person-years at risk. The relative risk of study outcomes of IAHI compared with LAIA was estimated by the Cox proportional hazard model with a robust sandwich variance matrix to account for the dependence of stable use sets within each subject^24^, and is presented as a hazard ratio (HR) and a 95% confidence interval (CI). The imbalanced baseline patient characteristics between the two study groups after matching were further adjusted in the multivariate Cox models. Subgroup analyses were performed by including the interaction terms of the drug group (i.e., IAHI versus LAIA) with the clinical characteristics of interest (i.e., prior history of CVDs, MVDs, and hospitalized hypoglycemia, age, gender, and diabetes duration) in the Cox models as covariates.

Four sensitivity analyses were conducted. First, we performed the intention-to-treat analysis, in which the occurrence of study outcomes, lost to follow-up in the NHI program, death, or the end of the database was considered as the censored point. Second, since the majority of study subjects were followed for less than 3 years, we restricted the study time horizon to a 3-year observational period to examine the relatively short-term clinical outcomes associated with study drugs. Third, a lag-time analysis was conducted to only consider clinical outcomes occurring at 30 days after the index date, with assuming that clinical events that had occurred within 30 days after the use of IAHI or LAIA would be less likely to be attributed to the drug effect. Fourth, to further enhance the between-group comparability after matching, matched sets with a difference in PS of > 0.1 were excluded. We further conducted the analyses based on a sub-cohort of patients without any prior history of CVDs or MVDs before the use of basal insulin to avoid the potential impacts of previous complications on the future development of vascular events and eliminate the concern of misclassification between underlying diseases and study outcomes. A two-tail *p*-value of less than 0.05 was considered statistically significant. All analyses were performed using SAS software version 9.4.

## Results

A total of 11,521 stable users (166,589 stable use sets) of IAHI and 37,651 stable users (661,434 stable use sets) of LAIA were identified. A final cohort with 8479 matched pairs of IAHI users and LAIA users was included for the analyses (Fig. 1). The average follow-up periods in the primary analysis under as-treated scenario were 0.8 and 1.5 years for the IAHI and LAIA groups, respectively; the follow-up times in the analysis under intention-to-treat scenario were 3.8 and 4.1 years for the IAHI and LAIA groups, respectively. Table 1 shows the baseline patient characteristics before and after the matching algorithm. Past GLA utilizations were statistically different between the study groups, with a SMD of > 0.1, before matching, but most characteristics were balanced after matching (expect for age at the index date, gender, hospital grade, and the history of retinopathy and cerebrovascular diseases). The PS distribution of the study groups before and after matching is presented in Supplementary Figure 2. This supports the enhancement of between-group comparability achieved by implementing our matching scheme. The variables which were statistically different between the study groups after matching were further adjusted in the Cox models.

Table 2 presents the results of the primary analyses on event rates and hazard ratios with 95% CIs of study outcomes. For the primary composite outcome of CVDs, there were 7.59 events per 1000 person-years and 4.56 events per 1000 person-years among IAHI and LAIA users, respectively. Compared with LAIA, IAHI use was associated with a significantly higher risk for composite CVDs (1.79; 1.20–2.67). The study results of individual CVD components are provided in Supplementary Table 2. For secondary outcomes, the use of IAHI compared with LAIA yielded significantly higher risks for three-point MACE (1.75; 1.01–3.04) and hospitalized hypoglycemia (1.82; 1.51–2.20), but significantly lower risks for composite MVDs (0.88; 0.84–0.91), nephropathy (0.90; 0.85–0.95), and retinopathy (0.85; 0.80–0.90). Risks for neuropathy (0.95; 0.88–1.02), all-cause mortality (1.72; 0.87–3.42), and fatal CVDs (1.16; 0.49–2.75) were similar between the groups.

**Table 2:** Primary analyses for the event rate and hazard ratio (95% confidence interval) of study outcomes for intermediate-acting human insulin versus long-acting insulin analogue (reference group).

Complications	Event rate per 1,000 person-years (no. of events)		Adjusted HR^c^ (95% CI)
	IAHI (n^b^ = 8479)	LAIA (n^b^ = 8479)	
**Composite CVDs**	7.59 (49)	4.56 (56)	1.79 (1.20–2.67)
**Three-point MACE**^a^	3.86 (25)	2.52 (31)	1.75 (1.01–3.04)
**Microvascular diseases**	1167.66 (4000)	1069.98 (5128)	0.88 (0.84–0.91)
Nephropathy	411.81 (2054)	309.94 (2656)	0.90 (0.85–0.95)
Retinopathy	408.90 (1983)	406.76 (3073)	0.85 (0.80–0.90)
Neuropathy	243.95 (1338)	172.81 (1720)	0.95 (0.88–1.02)
**Hospitalized hypoglycemia**	37.87 (240)	19.51 (236)	1.82 (1.51–2.20)
**All-cause mortality**	2.32 (15)	1.87 (23)	1.72 (0.87–3.42)
Fatal CVD	1.24 (8)	1.62 (20)	1.16 (0.49–2.75)

IAHI, intermediate-acting human insulin; LAIA, long-acting insulin analogue; HR, hazard ratio; CI, confidence interval; CVD, cardiovascular disease; MACE, major adverse cardiovascular events.

^a^Three-point MACE included non-fatal myocardial infarction, non-fatal stroke, and death due to cardiovascular diseases.

^b^ “n” refers to the number of stable use sets.

^c^The variables adjusted in these analyses were age, gender, hospital grade, history of retinopathy, and cerebrovascular disease, which are shown to be statistically different between IAHI and LAIA users at baseline (in terms of standardized mean difference value of > 0.1) in Table 1.

**Figure 2:**
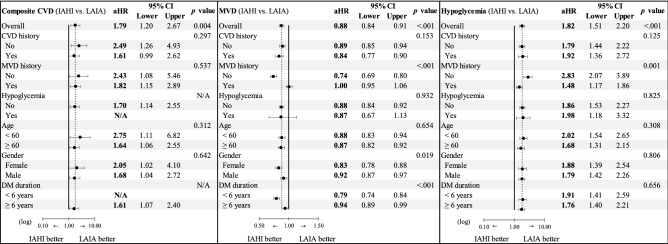
Primary and subgroup analyses for study outcomes of composite cardiovascular diseases, microvascular diseases, and hospitalized hypoglycemia for intermediate-acting human insulin versus long-acting insulin analogue (reference group). IAHI, intermediate-acting human insulin; LAIA, long-acting insulin analogue; aHR, adjusted hazard ratio; CVD, cardiovascular disease; MVD, microvascular disease; DM, diabetes mellitus; N/A, not available. (1) “N/A” indicates “not available”, indicating that the study event number was zero in any comparison subgroup and thus the analysis was not performed. (2) The variables adjusted in these analyses were age, gender, hospital grade, history of retinopathy, and cerebrovascular disease, which are shown to be statistically different between IAHI and LAIA users at baseline (in terms of standardized mean difference value of > 0.1) in Table 1.

Figure 2 shows the results of subgroup analyses. Patients without a history of MVDs, female patients, and patients with a diabetes duration of less than 6 years had a lower risk for composite MVDs associated with the use of IAHI versus LAIA, compared with their counterparties. In addition, a significant interaction between the use of IAHI versus LAIA and MVD history was found for hospitalized hypoglycemia. The sensitivity and sub-cohort analyses summarized in Table 3 show a trend of results similar to that obtained in the primary analyses (Table 2).

**Table 3:** Sensitivity and sub-cohort analyses for the event rate and hazard ratio (95% confidence interval) of study outcomes for intermediate-acting human insulin versus long-acting insulin analogue (reference group).

	Event rate per 1000 person-years (no. of events)		Adjusted HR^c^ (95% CI)
	IAHI	LAIA	
**Sensitivity analysis 1: intention-to-treat analysis**	(n^b^ = 8479)	(n^b^ = 8479)	
Composite CVDs	4.92 (158)	2.94 (103)	1.73 (1.35–2.23)
Three-point MACE^a^	3.19 (103)	1.90 (67)	1.73 (1.27–2.36)
Microvascular diseases	569.57 (6344)	657.86 (6988)	0.88 (0.85–0.91)
Nephropathy	182.47 (3947)	188.15 (4305)	0.93 (0.89–0.97)
Retinopathy	226.19 (4208)	273.15 (5063)	0.85 (0.81–0.88)
Neuropathy	116.58 (2737)	116.64 (2978)	0.97 (0.92–1.02)
Hospitalized hypoglycemia	27.29 (836)	25.33 (849)	1.06 (0.96–1.17)
All-cause mortality	2.62 (85)	1.56 (55)	1.68 (1.19–2.38)
Fatal CVD	1.29 (42)	0.76 (27)	1.78 (1.09–2.90)
**Sensitivity analysis 2: 3 years of maximum observational time**	(n^b^ = 8479)	(n^b^ = 8479)	
Composite CVDs	7.53 (46)	3.46 (36)	2.12 (1.35–3.32)
Three-point MACE^a^	3.92 (24)	1.34 (14)	2.68 (1.37–5.26)
Microvascular diseases	1188.53 (3978)	1116.59 (5049)	0.88 (0.84–0.91)
Nephropathy	423.51 (2021)	339.86 (2560)	0.89 (0.84–0.94)
Retinopathy	419.23 (1967)	434.59 (3000)	0.85 (0.80–0.90)
Neuropathy	252.57 (1322)	194.30 (1667)	0.94 (0.87–1.01)
Hospitalized hypoglycemia	38.09 (229)	19.87 (204)	1.81 (1.49–2.20)
All-cause mortality	2.45 (15)	0.48 (5)	5.02 (1.79–14.08)
Fatal CVD	1.31 (8)	0.19 (2)	6.18 (1.28–29.93)
**Sensitivity analysis 3: lag-time analysis**	(n^b^ = 8479)	(n^b^ = 8479)	
Composite CVDs	7.43 (48)	4.56 (56)	1.77 (1.18–2.64)
Three-point MACE^a^	3.71 (24)	2.52 (31)	1.70 (0.97–2.96)
Microvascular diseases	1001.51 (3742)	944.64 (4917)	0.90 (0.86–0.94)
Nephropathy	384.40 (1969)	293.92 (2573)	0.92 (0.87–0.98)
Retinopathy	354.65 (1783)	371.27 (2904)	0.84 (0.79–0.89)
Neuropathy	220.58 (1232)	164.60 (1655)	0.94 (0.87–1.01)
Hospitalized hypoglycemia	34.33 (218)	18.76 (227)	1.79 (1.48–2.17)
All-cause mortality	2.32 (15)	1.87 (23)	1.73 (0.87–3.42)
Fatal CVD	1.24 (8)	1.62 (20)	1.16 (0.49–2.75)
**Sensitivity analysis 4: excluding matched sets with a difference in propensity score of > 0.1**	(n^b^ = 7451)	(n^b^ = 7451)	
Composite CVDs	7.59 (45)	4.91 (53)	1.77 (1.17–2.67)
Three-point MACE^a^	3.70 (22)	2.78 (30)	1.64 (0.93–2.91)
Microvascular diseases	1104.57 (3483)	1061.14 (4510)	0.86 (0.82–0.90)
Nephropathy	386.91 (1777)	304.02 (2322)	0.90 (0.85–0.96)
Retinopathy	394.09 (1753)	409.54 (2724)	0.83 (0.78–0.88)
Neuropathy	1104.57 (3483)	1061.14 (4510)	0.86 (0.82–0.90)
Hospitalized hypoglycemia	35.58 (207)	18.81 (200)	1.83 (1.49–2.23)
All-cause mortality	2.02 (12)	2.03 (22)	1.53 (0.74–3.18)
Fatal CVD	1.18 (7)	1.85 (20)	1.53 (0.74–3.18)
**Sub-cohort analysis: excluding the patients with cardiovascular or microvascular disease history**	(n^b^ = 3779)	(n^b^ = 3779)	
Composite CVDs	2.84 (9)	0.56 (3)	3.99 (1.02–15.56)
Three-point MACE^a^	1.57 (5)	0.00 (0)	N/A
Microvascular diseases	493.36 (1074)	452.65 (1488)	0.90 (0.83–0.97)
Nephropathy	132.61 (375)	100.49 (487)	1.10 (0.96–1.26)
Retinopathy	257.70 (653)	247.19 (959)	0.86 (0.78–0.95)
Neuropathy	100.44 (292)	91.06 (441)	0.88 (0.76–1.03)
Hospitalized hypoglycemia	28.57 (89)	10.04 (53)	2.49 (1.76–3.53)
All-cause mortality	0.00 (0)	0.19 (1)	N/A
Fatal CVD	0.00 (0)	0.00 (0)	N/A

IAHI, intermediate-acting human insulin; LAIA, long-acting insulin analogue; HR, hazard ratio; CI, confidence interval; CVD, cardiovascular disease; MACE, major adverse cardiovascular events.

^a^Three-point MACE included non-fatal myocardial infarction, non-fatal stroke, and death due to cardiovascular diseases.

^b^“n” refers to the number of stable use sets.

^c^The variables adjusted in these analyses were age, gender, hospital grade, history of retinopathy, and cerebrovascular disease, which are shown to be statistically different between IAHI and LAIA users at baseline (in terms of standardized mean difference value of > 0.1) in Table 1.

## Discussion

To our knowledge, this is the largest population-based, prevalent new-user cohort study to investigate the association of the real-world use of basal insulins (IAHI versus LAIA) with vascular safety in patients with T2D. Our findings suggest that the use of IAHI versus LAIA in clinical practice may yield higher risks for CVDs and hospitalized hypoglycemia but a lower risk for MVDs. In addition to patients with cost concerns, IAHI may be preferable for patients without a history of MVDs, female patients, and patients with a diabetes duration of less than 6 years to reduce the risk for MVDs. However, it is crucial to optimize the prevention of adverse CVD outcomes, and the monitoring and management of hypoglycemia. LAIA may be preferable for patients who are more vulnerable to hypoglycemia or at higher risks for CVDs.

There are no published randomized controlled trials that have assessed the comparative vascular effects of basal insulin. The Outcome Reduction with an Initial Glargine Intervention (ORIGIN) trial investigated the clinical outcomes of basal insulin glargine compared with standard care and reported a comparable cardiovascular effect between insulin glargine versus standard care^25^. However, the direct comparison of the results between the present study and the ORIGIN trial should be cautious due to differences in the study cohorts and comparison groups between two studies. First, the present study included a real-world T2D population of diverse demographic and clinical characteristics, whereas the ORIGIN trial only targeted the patients at high risk of vascular diseases with either prediabetes or early T2D. Further, we specified the IAHI as the comparator group, while the ORIGIN trial employed the standard care group as the comparator. As expected, our study findings were not fully comparable with the results of the ORIGIN trial.

A few retrospective cohort studies have analyzed incident new users of basal insulin in a T2D population who were free of vascular complications at baseline; inconclusive results were reported. Juhaeri et al. identified a cohort of 65,619 T2D patients with newly initiated basal insulin and without a history of heart failure (HF), stroke, or acute myocardial infarction (AMI) from a U.S. claims database. Comparable incidence rates of composite CVD events were found between the LAIA group (insulin glargine) and the IAHI group (including ultralente, lente, or NPH insulin), except for a lower rate of AMI found in the LAIA group. However, the residual confounding bias due to unmeasured variables (i.e., diabetes severity and previous GLA use) is a major concern for the interpretation of these study results^8^.

Hall et al. evaluated the risks of macro- and microvascular events in 3,427 patients with T2D who were newly prescribed with LAIA or NPH, stratified by the utilization pattern of baseline oral GLAs (i.e., two or three oral GLAs). Comparable incidence rates of macrovascular complications were found between the study groups. Although there was a significantly increased risk of microvascular complications in the NPH group compared to the LAIA group among patients treated with three oral GLAs at baseline, this finding was not confirmed in the sensitivity and subgroup analyses^9^.

Cammarota et al. analyzed the administrative data from Italy and reported that compared to LAIA (insulin glargine), the use of NPH was associated with a significantly higher risk of CVDs but a non-significantly different risk of MVDs^10^. However, some limitations inherent to this study should be noted, including a small sample size (403 matched pairs of NPH and LAIA users), a short observational period (maximum of 3 years), unavailable information about diabetes duration, and a mixed study cohort where type 1 diabetes and type 2 diabetes patients could not be differentiated, which together affect the validity of the study results.

Recently, Neugebauer et al. included 127,600 insulin-naïve adults with T2D at the 4 U.S. health care delivery systems to examine the association of using human insulin only (HI group) versus analogue insulin with or without human insulin (AI group) with mortality and major cardiovascular events. They found similar risks of MI, hospitalization for HF, stroke or cerebrovascular accident, CVD mortality, or overall mortality between the HI and AI groups. However, the use of short-/rapid-acting and/or long-acting insulin products was not differentiated, implying that the insulin users of basal-only, basal‐plus, basal‐bolus or premixed insulin regimens were all pooled in the analyses. Our study cohort that did not consider the users exclusively on a premixed insulin regimen or bolus insulin treatment would represent a sub-population of this previous study^26^.

Furthermore, different from previous studies using the incident new-user cohort design to only include the naïve users of basal insulin and those free of vascular complications before insulin initiation, our study included a broad spectrum of real-word adults with T2D being treated with basal insulin through the prevalent new-user cohort design to enhance the generalizability of the study findings. The increased risk of hypoglycemia associated with the use of IAHI versus LAIA found in our study is consistent with the findings in previous studies^2–5^. An increasing body of evidence suggests that hypoglycemia may contribute to the development of CVDs in the T2D population^27,28^. Our previous study of insulin therapy in T2D supports that the occurrence of hypoglycemia plays an independent role in the risk of developing CVDs in this population^11^. Therefore, compared to LAIA users, the increased risk of hypoglycemia in IAHI users observed in this study may partially explain the higher risk of CVDs. Moreover, the lower risk of MVDs associated with IAHI versus LAIA shown in this study might be explained by the better glucose-lowering effects of IAHI based on its pharmacokinetic profiles as revealed in past studies^3,7^, although some studies showed comparable glycemic control between IAHI and LAIA^2,6^.

Compared to previous studies^8–10^, the present study has several strengths. First, considering that insulin therapy in clinical practice is often initiated in the later treatment course of diabetes due to clinical inertia, most patients may have been treated with multiple GLAs and had vascular complications at the initiation of basal insulin. Considering this, previous studies that utilized the incident new-user design for including insulin-naïve patients free of vascular complications at baseline restricted study cohort to those who were under-represented basal insulin users in the real world. Instead, we implemented the prevalent new-user cohort design to include a board spectrum of T2D patients being treated with basal insulin and with or without baseline vascular complications, which ensured comprehensive assessment and enhanced the external validity of the study results to the real-world diverse T2D population requiring basal insulin therapy. Second, to minimize potential biases resulting from the inclusion of all possible real-world basal insulin users in the analyses (i.e., time-related biases due to different initiation periods of basal insulin, confounding from variations in diabetes severity and past GLA use), we performed a rigorous three-step matching to achieve a greater level of comparability between the study groups, as evidenced by most baseline patient characteristics having SMD values of < 0.1 (Table 1). This matching scheme ensures the internal validity of the study estimates. Third, we restricted the study cohort to those who were stably treated with basal insulin and performed the primary analyses under the as-treated scenario. These might mitigate the potential biases due to the inclusion of short-term or accidental users of study drugs and non-adherence problems during follow-up. Lastly, considering the diverse circumstances in real-world settings, we conducted a series of subgroup and sensitivity analyses to corroborate our findings in primary analyses. This strengthens the confidence of the result interpretation and the implications of our findings to facilitate real-world clinical decisions.

Several limitations should be acknowledged. First, we applied a rigorous matching scheme to control for patient characteristics between the study groups, but, like studies using administrative claims data, the residual effects attributable to unmeasured confounders (e.g., laboratory data, physicians’ prescribing preferences, or patients’ health behaviors) may not be fully avoided. Specifically, the variability of baseline blood glucose levels a patient has could influence physicians’ choice of insulin therapy (i.e., IAHI that can be administered more flexibly for better control of blood glucose fluctuations may be preferred to patients with a greater variability of blood glucose levels). Also, patients’ health behaviors may affect physicians’ prescribing decisions. For example, IAHI that is generally required to be injected multiple times in a day and has a higher risk for hypoglycemia side effect may be more likely to be given to patients with better medication adherence, and self-awareness and management for hypoglycemia. Nevertheless, efforts have been made to minimize the residual confounding effects through the adjustment for a comprehensive list of baseline variables to ensure the duration and severity of T2D, the use of GLA regimens and CVD-related medications, and the status of comorbidities and complications being comparable between study groups after the matching. Although a prospective randomized controlled trial can overcome the potential confounding by measured or unmeasured covariates, there may be less motivation for initiating a long-term, costly clinical trial to evaluate the vascular outcomes of a basal insulin regimen with IAHI versus LAIA. Second, due to the small number of mortality events, this study may be underpowered to detect a significant association of IAHI versus LAIA with all-cause mortality or fatal CVDs; future studies are thus warranted. Third, the operational definitions of some study outcomes (i.e., fatal CVD, MVDs) have not been validated in the NHIRD yet, and therefore, the absolute numbers of clinical events in each study group might not be completely and accurately ascertained. However, this might less affect our main conclusions which were based on the analytic results of relative hazards of clinical events between two study groups. Fourth, the interpretation of our results may be limited to a population with T2D under a healthcare system with universal health insurance coverage, where the co-pay of IAHI or LAIA would not be a serious concern for patients themselves in the clinical decision of selecting the type of basal insulin. However, the costlier LAIA (versus IAHI) still has a significant economic impact from the health system and social perspectives.

In summary, this real-world cohort study consisting of a diverse T2D population provides evidence on the long-term vascular safety of basal insulins. Our findings have important therapeutic implications—in usual practice for T2D patients, strategies for minimizing the risks of CVDs and hospitalized hypoglycemia should be aware and optimized for the use of a basal insulin with IAHI versus LAIA, while IAHI may be associated with a lower risk of MVDs. More studies are needed to confirm the findings from the present study.

## Supplementary Information

Supplementary Information.
