# Supplementary material for: A nationwide cohort study for comparative vascular safety of long-acting insulin analogue versus intermediate-acting human insulin in type 2 diabetes
Source: Sci Rep. 2021 Feb 18;11:4152. doi: 10.1038/s41598-021-83253-6 (PMC7893071; doi:10.1038/s41598-021-83253-6)
Supplement: Supplementary file 1 — Supplementary Information. [file 41598_2021_83253_MOESM1_ESM.docx]

**Original Article**

A nationwide cohort study for comparative vascular safety of long-acting insulin analogue versus intermediate-acting human insulin in type 2 diabetes

Chun-Ting Yang, MS^1,†^

Kuan-Ying Li, MS^1,†^

Chen-Yi Yang, MS^1^

Huang-Tz Ou, PhD^1,2,3*^

Shihchen Kuo, RPh, PhD^4^

**Author Affiliations:**

^1^Institute of Clinical Pharmacy and Pharmaceutical Sciences, College of Medicine, National Cheng Kung University, Tainan, Taiwan

^2^Department of Pharmacy, College of Medicine, National Cheng Kung University, Tainan, Taiwan

^3^Department of Pharmacy, National Cheng Kung University Hospital, Tainan, Taiwan

^4^Division of Metabolism, Endocrinology & Diabetes, Department of Internal Medicine, University of Michigan Medical School, Ann Arbor, MI, United States

^†^These two authors contributed equally.

***Corresponding Author:**

Huang-Tz Ou, PhD

Institute of Clinical Pharmacy and Pharmaceutical Sciences

College of Medicine, National Cheng Kung University

1 University Road

Tainan 701, Taiwan

Telephone: 886-6-2353535 ext.5685

Fax: 886-6-2373149

Email: [huangtz@mail.ncku.edu.tw](mailto:huangtz@mail.ncku.edu.tw)

1. **Supplementary Figure 1**: Three-step matching algorithm
2. **Supplementary Figure 2**: The propensity score (PS) distribution of IAHI and LAIA stable use sets before and after PS matching
3. **Supplementary Table 1**: International Classification of Diseases, Ninth Revision, Clinical Modification (ICD-9-CM) codes for defining study outcomes
4. **Supplementary Table 2**: Primary analyses for the event rate and hazard ratio (95% confidence interval) of individual components of cardiovascular outcomes for intermediate-acting human insulin versus long-acting insulin analogue (reference group)

Supplementary Figure 1. Three-step matching algorithm


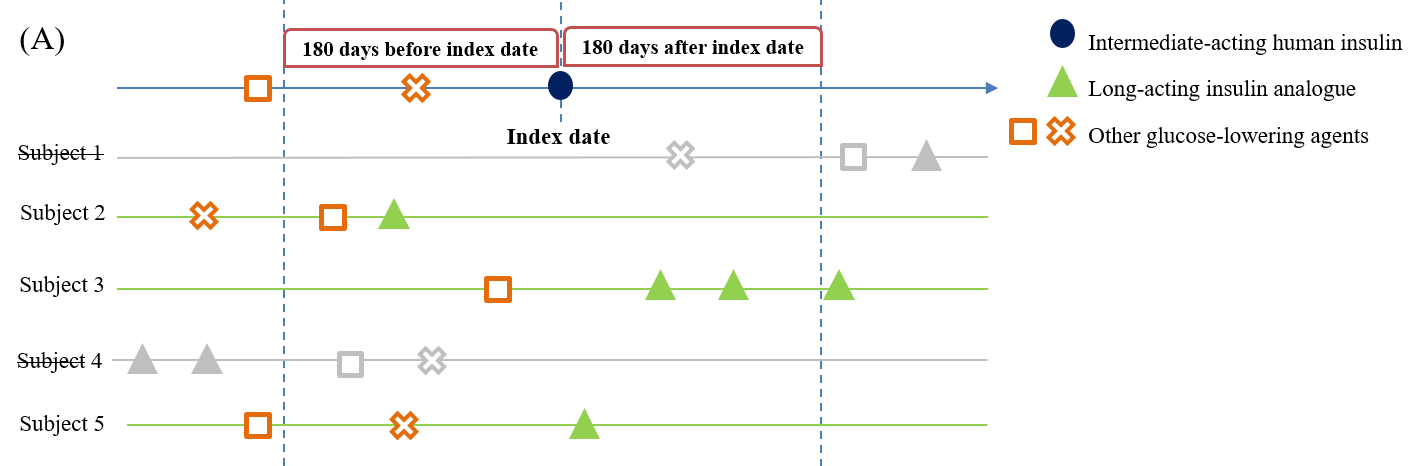


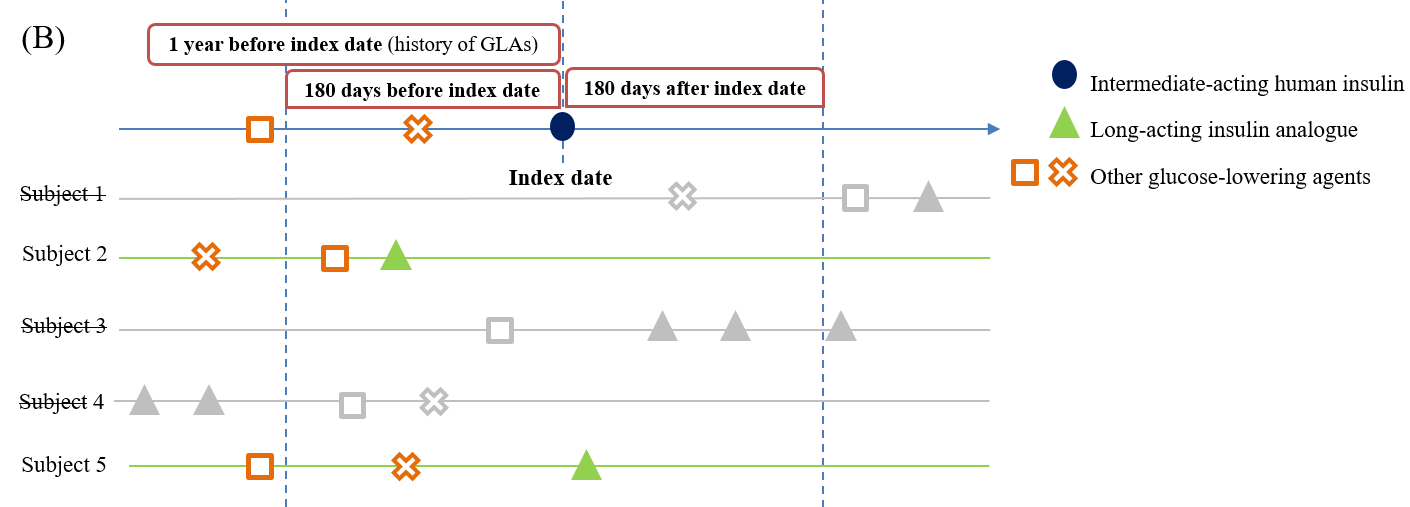


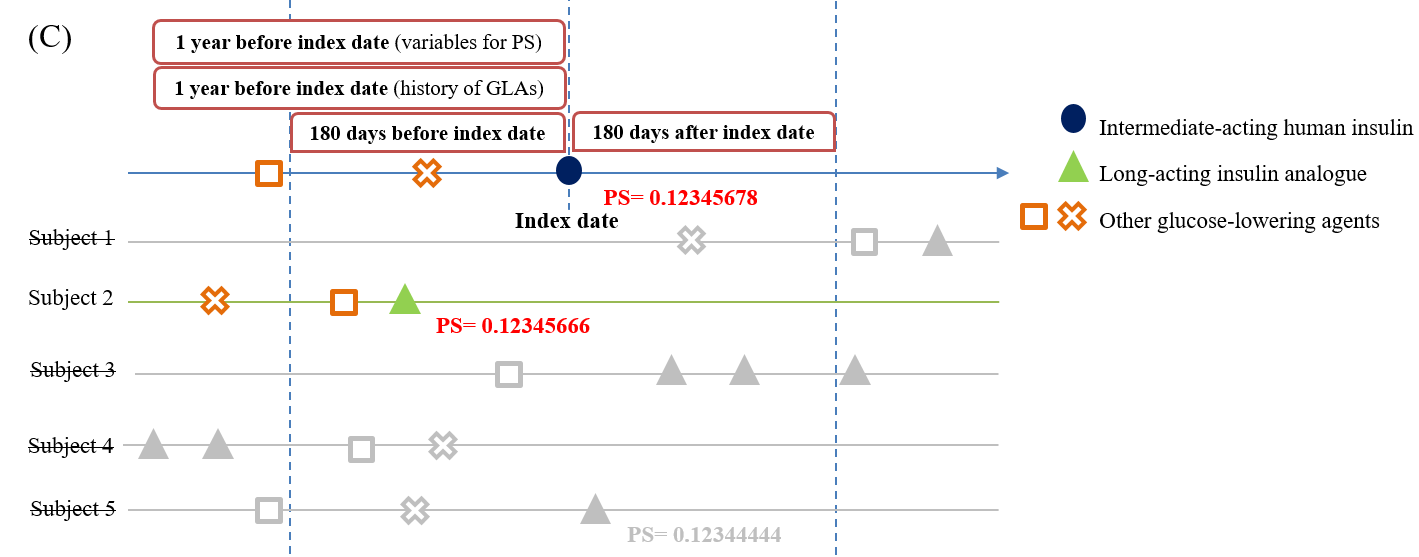


1. The first step was to match the index date with the pre-defined time interval (i.e., long-acting insulin analogue [LAIA] stable use set with an index date falling within ±180 days of the index date of the intermediate-acting human insulin [IAHI] stable use set). For example, there were three subjects (Subject 2, 3, 5) that used LAIA being matched for the subject that used IAHI. Subject 1 and Subject 4 were excluded because their index dates of using LAIA were not in 180-day time interval.
2. The second step was to match the previous utilization patterns of glucose-lower agents (GLAs). Those who exposed to the same GLA classes (e.g., GLA “□” and “Ｘ” of the IAHI stable use set), with a maximum 90-day drug supply difference (±45 days) for each specific GLA in the previous 1 year of the index date were matched. In detail, for the IAHA stable use set who had been exposed to the GLA “□” (with a 270-day drug supply) and the GLA “Ｘ” (with a 180-day drug supply) in the 1-year baseline period, only the stable use sets of LAIA who met the criteria of “having exposure to both the GLA”□” (with a total of drug supply ranged from 225-315 days) and the GLA“Ｘ” (with a total of drug supply ranged from 135-225 days) in the 1-year baseline period” would be matched. Subject 3 was excluded due to the different utilization patterns of GLAs prior to using LAIA.
3. The third step was to match the propensity score based on baseline subject characteristics measured from the year before or at the index date. The propensity score of Subject 2 was closest to that of the study drug subject, and thus Subject 2 was selected as the matched IAHI subject.

Supplementary Figure 2. Distribution of propensity scores (PS) of intermediate-acting human insulin (IAHI) and long-acting insulin analogue (LAIA) stable use sets before and after PS matching


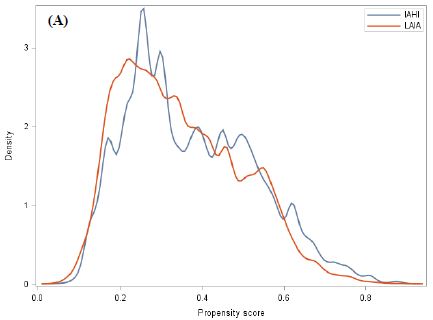


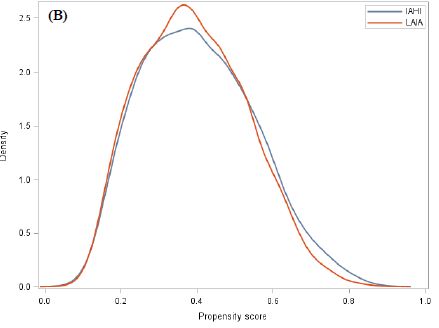


1. The PS distribution of IAHI and LAIA stable use sets before PS matching (i.e., the 3^rd^ matching step)
2. The PS distribution of IAHI and LAIA stable use sets after PS matching

Supplementary Table 1. International Classification of Diseases, Ninth Revision, Clinical Modification (ICD-9-CM) codes for defining study outcomes

|  | ICD-9-CM disease codes | ICD-9-CM procedure codes |
| --- | --- | --- |
| Cardiovascular diseases (identified from inpatient and emergency department records) | | |
| Acute myocardial infarction | 410 |  |
| Ischemic heart disease | 411, 413, 414, V45.81, V45.82 | 36.0, 36.1, 36.2, 36.3, 36.9, 88.5, 00.66 |
| Heart failure | 428 |  |
| Stroke or transient ischemic attack | 430-437, V12.54 | 00.61, 00.63, 38.11, 38.12 |
| Cardiogenic shock | 785.51 |  |
| Sudden cardiac arrest | V12.53 |  |
| Arteriosclerotic cardiovascular disease | 429.2 |  |
| Arrhythmia | 426, 427 |  |
| Microvascular diseases (identified from inpatient and outpatient department records) | | |
| Nephropathy | 580, 581, 582, 583, 585, 586, 250.4, 593.9 | 38.95, 39.27, 39.42, 39.95, 54.98, 55.4, 55.5, 55.6 |
| Retinopathy | 361, 362, 369, 250.5, 379.23 | 12.41, 12.73, 14.23, 14.24, 14.25, 14.33, 14.34, 14.35, 14.53, 14.54, 14.55, 16.92, 16.99 |
| Neuropathy | 354, 355, 458, 250.6, 356.9, 358.1, 951.0, 951.1, 951.3, 713.5, 357.2, 337.0, 337.1, 564.5, 536.3, 596.54 |  |
| Hypoglycemia (identified from inpatient and emergency department records) | 251.0, 251.1, 251.2, 270.3, 962.3 |  |
| Death | 1. Recorded as death cases (i.e., Tran_code = 4) or discharged due to terminal state (i.e., Tran_code = A) in the inpatient department files. 2. No further records in Taiwan’s National Health Insurance Program after 30 days of being recorded as death cases. | |
| Fatal CVD | Having cardiovascular diseases (defined by the abovementioned ICD-9-CM disease codes and procedure codes) occurred in the 90 days prior to death. | |

Supplementary Table 2. Primary analyses for the event rates and hazard ratios (95% confidence intervals) of individual components of cardiovascular outcomes for intermediate-acting human insulin versus long-acting insulin analogue (reference group)

| **Complications** | **Event rate per 1,000 person-years** (no. of events) | |  | **Adjusted HR**^‡^ (95% CI) |
| --- | --- | --- | --- | --- |
|  | **IAHI** (n^†^=8,479) | **LAIA** (n^†^=8,479) |  |  |
| Myocardial infarction | 1.24 (8) | 1.54 (19) |  | 1.39 (0.58-3.30) |
| Stroke | 2.01 (13) | 0.81 (10) |  | 2.19 (0.94-5.10) |
| Heart failure | 2.94 (19) | 2.60 (32) |  | 1.40 (0.77-2.54) |
| Ischemic heart disease | 3.71 (24) | 3.09 (38) |  | 1.42 (0.83-2.43) |
| Atherosclerotic cardiovascular disease | 0 (0) | 0 (0) |  | Not applicable |
| Arrhythmia | 1.08 (7) | 0.89 (11) |  | 1.39 (0.53-3.70) |
| Cardiogenic shock | 0.15 (1) | 0 (0) |  | 0.85 (0.80-0.90) |
| Sudden cardiac arrest | 0 (0) | 0 (0) |  | Not applicable |
